# Autoregulation in Resistance Training: Addressing the Inconsistencies

**DOI:** 10.1007/s40279-020-01330-8

**Published:** 2020-08-19

**Authors:** Leon Greig, Ben Hayden Stephens Hemingway, Rodrigo R. Aspe, Kay Cooper, Paul Comfort, Paul A. Swinton

**Affiliations:** 1 School of Health Sciences, Robert Gordon University, Garthdee Road, Aberdeen, UK; 2 Directorate of Psychology and Sport, University of Salford, Frederick Road, Salford, Greater Manchester UK; 3 Institute for Sport, Physical Activity and Leisure, Carnegie School of Sport, Leeds Beckett University, Leeds, UK; 4 Centre for Exercise and Sport Science Research, Edith Cowan University, Joondalup, Australia

## Abstract

Autoregulation is a process that is used to manipulate training based primarily on the measurement of an individual’s performance or their perceived capability to perform. Despite being established as a training framework since the 1940s, there has been limited systematic research investigating its broad utility. Instead, researchers have focused on disparate practices that can be considered specific examples of the broader autoregulation training framework. A primary limitation of previous research includes inconsistent use of key terminology (e.g., adaptation, readiness, fatigue, and response) and associated ambiguity of how to implement different autoregulation strategies. Crucially, this ambiguity in terminology and failure to provide a holistic overview of autoregulation limits the synthesis of existing research findings and their dissemination to practitioners working in both performance and health contexts. Therefore, the purpose of the current review was threefold: first, we provide a broad overview of various autoregulation strategies and their development in both research and practice whilst highlighting the inconsistencies in definitions and terminology that currently exist. Second, we present an overarching conceptual framework that can be used to generate operational definitions and contextualise autoregulation within broader training theory. Finally, we show how previous definitions of autoregulation fit within the proposed framework and provide specific examples of how common practices may be viewed, highlighting their individual subtleties.

## Key Points

**Table:** 

Autoregulation is described by an emergent process that can be used to systematically individualise physical training. This is achieved through a flexible framework that enables practitioners to continually adjust training programmes over time based on measurement of an individual’s performance.
Despite substantial developments since the 1940s, the lack of an overarching framework has led to inconsistencies in definitions and terminology used throughout associated research and practice. This has led to an ambiguity surrounding how best to implement a range of autoregulation strategies in practice, and a lack of synthesis within research.
Future research should focus attention on identifying key features of the measurement and adjustment process that can be used to identify and define general autoregulatory principles and/or guidelines.

## Introduction: Autoregulation of Training, Perceived Benefits, and Continued Development

**Fig. 1:**
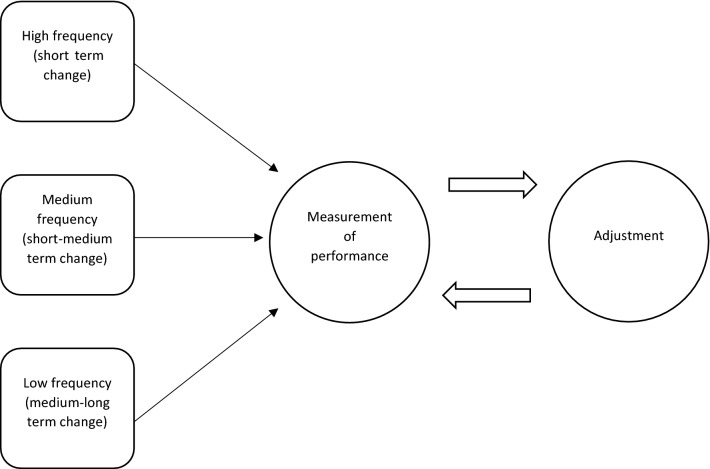
Autoregulation of training viewed as a continuous two-step feedback process

The concept of individualisation is widely accepted within sport and exercise science [1]. Following this perspective, it is commonly believed that purposefully adjusting training to coincide with measurements of an individual’s response to training- and non-training-related stressors (e.g., sleep, nutrition, and illness) can both maximise increases in performance and deter the onset of maladaptive symptoms such as injury and overtraining [2, 3]. In practice, this individual response is frequently estimated by measuring performance in one or more tests thought to assess the physical quality (e.g., strength, power, and aerobic capacity) being trained [4]. The general concept that training should be adjusted in accordance with measurements of an individual’s performance (and potentially perceptions of ability to perform) is referred to as autoregulation [5]. Here, the prefix ‘auto’ refers to regulation based on measurements made on the individual being measured, and not to highlight that the process is required to follow automated rules. At present, two broad implementations of autoregulation are presented in research. The first and most prevalent approach is to measure and adjust training daily [6] to reflect high-frequency fluctuations in performance that may be caused by both training- and non-training-related stressors. In contrast, the second approach measures and adjusts training on a less frequent basis (e.g., weekly or at the end of monthly short training blocks) to reflect more chronic changes in performance that are caused primarily by training-related adaptations in both central and peripheral systems [7]. Whilst researchers have focused on these two common approaches in isolation, a combination of the two may be implemented in practice to encourage a more continuous adjustment of training that better responds to the changing dynamics of an individual [6]. Based on the range of approaches available to researchers and practitioners, a more general perspective is to view autoregulation as a malleable training framework (Fig. 1) that permits systematic adjustment of training variables based primarily on the assessment of an individual’s performance [6].

A growing evidence base indicates that the autoregulation of training may be superior to well-designed training regimes that feature predetermined loading strategies for targeting physical qualities such as strength [7–9] and accretion of lean body mass [10]. Where increased effectiveness due to autoregulation has been observed, hypotheses relate to a closer match between the intended and delivered training stimulus on a session by session basis and/or at the programme level [11]. These hypotheses generally stem from the observation that in the traditional approaches to exercise programming, longitudinal blocks of training are prescribed using a singular baseline measure of performance taken prior to the beginning of a training cycle (e.g., 1RM testing prior to a strength phase) [12]. While this approach enables practitioners to prescribe training with some degree of individualisation [13], researchers have argued that the fixed nature across time may lead to periods of sub-optimal loading [14]. Periods of mismatch between the desired training stimulus and that which is received may be due to both day to day fluctuations in an individual’s performance and short-term adaptations causing improvements to be substantially greater (or lesser) than expected [15]. It has, therefore, been suggested that adjusting the training received to coincide with more current estimations of an individual’s performance—as is the case with autoregulation—may be advantageous to ensure sessions more closely align with an individual’s current performance level and the overarching training goals [16].

Whilst the main purported advantages of autoregulation relate to a better alignment of the intended training stimulus from a physiological standpoint, it has also been suggested that the framework may enhance psychological outcomes such as exercise adherence [17]. For example, some autoregulation practices enable individuals to self-select their training sessions from a predetermined pool based on their perceived capability to perform [8, 9]. It has been suggested that these variants of autoregulation may facilitate greater programme adherence and enjoyment due to increased autonomy [18]. To date, however, these latter hypotheses have yet to be systematically investigated and comparable methods of autoregulation have received limited study with research confined primarily to weightlifting [9], cycling [19], and powerlifting [8].

Whilst the general concept of autoregulation of training was introduced in the 1940s [20], only recently has a range of novel implementation methods begun to emerge [21]. Developments have occurred due to both an increased awareness of the advantages of ongoing monitoring in both sport and health settings, as well as improved technologies enabling logistically feasible, accurate, and reliable measurement of physiological, performance, and perceptual data [4, 22]. Most developments in the autoregulation of training have occurred in sporting contexts where a culture of data collection is widespread, and practitioners routinely seek innovative methods to optimise performance [23]. Additionally, more novel measurement technologies are frequently emerging in sporting contexts, enabling practitioners to individually tailor training regimes using, for example, variables such as heart rate variability [24], blood and salivary biomarkers [25, 26], as well as perceptual measures of well-being and stress [27]. Whilst these measures are gaining increasing recognition as athlete monitoring tools [22], it is still relatively unclear how they relate to an individual’s performance and whether they can be used to effectively adjust training. Additionally, whilst autoregulation and the subsequent individualisation of training may be more effective as a training strategy in health settings due to the extreme heterogeneity commonly observed (i.e., medical history, disease severity, and additional treatment regime), research regarding the implementation of autoregulation and other novel exercise frameworks in the health domain is limited [28]. Therefore, the current review will focus on the settings and methods most used in autoregulation research and practice. At present, this includes resistance training of athletes employing objective and subjective measurements of performance including examples such as countermovement jump (CMJ) kinematic and kinetic variables [29], barbell velocity [12], and measurements associated with rating of perceived exertion (RPE) [30]. Briefly, the collection and assessment of these variables represent the majority of research in the area and are commonly used to adjust both training intensity and volume [6, 16] over a range of timescales.

## The Development of Autoregulation of Training

In this section, a brief chronological overview of autoregulation practices in resistance training is provided to outline the development and scope of strategies currently employed. DeLorme [20] is frequently credited with the initial development of autoregulation as a training framework in the 1940s [5]. DeLorme [20] observed in rehabilitation settings that adjusting the exercise load based on weekly performance of a ten-repetition maximum (RM) test resulted in superior improvements during a strength training programme compared to the traditional methods where increments in load were fixed. In 1979, Knight revised the work of DeLorme to include daily modifications in the load lifted based on an adjustment table (Table 1). Knight [31] proposed that more frequent adjustments were required to account for the consistent fluctuations in performance commonly observed during rehabilitation, as well as the highly individualised rate of strength progression [32]. The daily adjustable progressive resistive exercise (DAPRE) protocol developed by Knight [31] measured performance during the penultimate set of an exercise to momentary muscular failure at an estimated 6 RM load (Table 2). This performance was then used to adjust the resistance for the fourth and final set that would then be used to represent the new 6 RM estimate for the following session [33]. Since it first received empirical support [34], Knight’s DAPRE protocol has gained popularity in physiotherapy practice [35], and remains a popular autoregulation protocol in the treatment of injuries and pathologies of the knee [35, 36].

**Table 1:** Adjustment table guidelines adapted from Knight [31], with permission

Number of repetitions performed	Adjustment required	
	Fourth set^a^	Next session^b^
≤ 2	Decrease 5–10 lb	Decrease 5–10 lb
3–4	Decrease 0–5 lb	Keep the same
5–6	Keep the same	Increase 5–10 lb
7–10	Increase 5–10 lb	Increase 5–15 lb
> 10	Increase 10–15 lb	Increase 10–20 lb

*RM* repetition maximum

^a^Adjustment in weight is based on performance during the third set

^b^Performance in the fourth set is used as the new 6RM estimate to prescribe load in the following session

1 lb = 0.45 kg

**Table 2:** Characteristics of the original DAPRE protocol outlined by Knight [31]

Set	Percentage of 6RM estimate (%)	Repetitions performed
1	50	10
2	75	6
3	100	AMRAP
4	Adjustment	AMRAP

*AMRAP* As many repetitions as possible, *DAPRE* daily adjustable progressive resistive exercise, *RM* repetition maximum

Despite the success of the DAPRE protocol in rehabilitative settings, the training generated from the framework was perceived as monotonous and unlikely to transfer to broader training goals [35]. In 2000, Siff attempted to increase the scope of DAPRE by including 3, 6, and 10 RM variants that could be applied to target a range of physical qualities (power, strength, and hypertrophy) in a periodized manner, matching those commonly used by athletes [37]. Siff [37] was also the first author to popularise the term autoregulation when referring to training adjustments based on measurement of performance and is generally credited as being the first to apply autoregulation outside a rehabilitation setting.

As the concept of autoregulation grew in practice, it was recognised that frequently measuring performance through tests to momentary muscular failure may be unnecessary and potentially detrimental due to increased fatigue [5]. Therefore, alternative methods of autoregulation were introduced into the scientific literature. McNamara and Stearne [9] investigated the use of Kraemer and Fleck’s [38] flexible nonlinear periodised (FNLP) model as an autoregulation strategy with inexperienced resistance trained males and females. The training practice enabled each individual to self-select their daily exercise session (either 10, 15, or 20 RM) based on their perceived performance capability [9] across each 4-week mesocycle, before completing the next block of training. The study compared performance increases over a 12-week intervention with a volume and intensity matched group that performed training sessions in a predetermined order. The FNLP group demonstrated significantly greater improvements in leg press 1 RM; however, they failed to show greater improvements in either chest press 1 RM or standing long jump. The authors concluded that there was strong theoretical support for the novel method and that the large difference in average improvements in leg press (62 vs. 16 kg) observed between the groups also provided empirical support. The extent to which these results were influenced by the inexperienced nature of the participants remains unclear; however, more recent research with well-trained participants showed no performance benefits with FNLP compared with a matched training program not featuring autoregulation when participants were allowed to self-select the order of their exercise sessions (strength, power, or hypertrophy) on a week-by-week basis [8].

Following the study by McNamara and Stearne [9], both research and practice have focused predominantly on two of the most popular methods of autoregulation within resistance training: velocity-based training (VBT) [5] and perceptual measures of exertion [30]. The central premise of VBT is that the resistive load can be prescribed and manipulated with velocity ranges which target a desired physical quality, rather than as a percentage of a pre- determined 1RM. It has been hypothesised that this practice could account for daily fluctuations in performance and thereby enhances the overall training stimulus [39]. Recent studies provide support for an enhanced training stimulus with results demonstrating superior improvements in both maximal strength and CMJ performance for individuals following a VBT protocol compared to those following a percentage-based programme [12, 40]. However, to perform VBT, a device is required to accurately measure barbell velocity [41] and the accuracy of some devices have been shown to be limited [42–44]. In contrast, more accessible methods of autoregulation have been developed that do not require the use of sophisticated measurement devices. For example, Zourdos et al. [30] introduced and subsequently validated a modified RPE scale that has been used to regulate both the intensity and volume of exercise based on an athlete’s perceived readiness [30]. To date, this method remains one of the most prevalent autoregulation strategies and has been shown to be an effective tool for multiple participant groups [16].

Based on the brief overview above, it is clear that autoregulation of training has undergone substantial development since the 1940s; however, several limitations still exist. For example, while there is a general consensus that autoregulation can be defined as a training framework that permits the systematic adjustment of specific training variables (load, volume, and organisation) in line with daily and or weekly measurements of performance [5–7, 21], contradictions still exist in the description and use of key concepts and terminology. For example, readiness is a term that is often used to describe the phenomenon of short-term fluctuations in performance, or the perceived ability to perform exercise [21]. In this context, readiness is viewed as a means of autoregulating the training received based on changes in performance that are unanticipated and due primarily to non-training-related factors [28, 30]. In contrast, some authors have used readiness to refer to anticipated changes in performance that are more closely related to concepts such as adaptation and fatigue [45]. In addition, constructs such as adaptation [7, 30, 46], readiness [16, 47], and fatigue [45] are frequently used interchangeably when discussing the autoregulation of training. This has led to ambiguity and confusion regarding the relatedness of these constructs and whether adjustments to training should be made on the basis of one, multiple or all of these. As a result, there is a need for clear operational definitions that facilitate consistency amongst both researchers and practitioners, such that evidence can be better synthesised and evaluated. Moreover, a clear theoretical framework that identifies the most relevant features of the measurement and adjustment processes within autoregulation is required. Finally, refinement of key concepts and the overall structure of autoregulation will assist in identifying contemporary practices that may at first appear disparate, but can ultimately be viewed under the same framework, enabling more central questions to be addressed. Therefore, the purpose of this review was twofold. First, we sought to derive a conceptual framework that could be used to operationally define key constructs of autoregulation of training and enhance the consistency of future research. Second, we aimed to provide a brief discussion of how both historic and contemporary autoregulation methods may be contextualised by the framework presented herein to supplement its integration in practice.

## Developing an Autoregulation Framework

In the following sections, we specify an autoregulation framework that provides operational definitions for the key constructs required in the measurement of performance. The framework builds upon the popular fitness–fatigue model (FFM) to link training to performance. We also show how previous definitions and conceptions of autoregulation can be made consistent with the framework proposed. Additionally, we provide model examples to highlight subtleties in the framework and its application in practice.

### Introduction to the Fitness–Fatigue Model

**Fig. 2:**
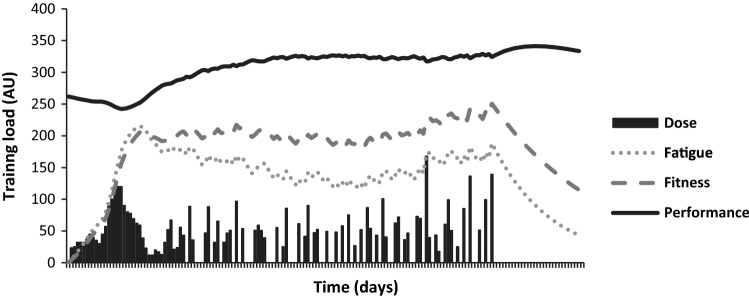
Modelled performance change over a training block highlighting the influence of fitness and fatigue after-effects on resultant performance. *AU* Arbitrary units

The FFM is arguably the most influential model used to conceptualise the physical training process [48]. Originally developed by Banister et al. [49], the underlying principles of the FFM have become thoroughly engrained in sport and exercise science and provide the basic rationale behind a large body of contemporary practice [48]. In its most basic form, the FFM posits that a single bout of training creates two antagonistic after-effects including a long-lasting and low-magnitude positive fitness effect, and a negative short-lasting and high-magnitude fatigue effect [50]. Performance on a given day is, therefore, said to equal some baseline measure of performance plus the sum of the fitness and fatigue effects generated from all previous training sessions (Fig. 2). The FFM can also be viewed from a dose–response perspective, such that larger doses of training are required to produce greater changes in performance.

Mathematical implementation of the FFM is described by a differential equation tailored to each individual, linking the training dose received (input) to a performance measure (output, e.g., vertical jump height, bench press RM, 40 m sprint time). Tailoring of the FFM is achieved by setting parameters in the equation to match the magnitude and decay rates of both the positive and negative after-effects experienced by the individual [50]. Since the model’s original conception [49], multiple researchers have proposed updates to better reflect various empirically observed features of the training process (e.g., the residual effects of previous training sessions on future training). Whilst a number of limitations still exist (readers are referred to comprehensive reviews by Hellard [51] and Pfeifier [52]), the rich conceptual nature of the model and capability to derive actual predictions of performance, make the FFM a suitable candidate for developing an autoregulation framework with operationally defined constructs.

### Using the Fitness–Fatigue Model to Operationalise Autoregulation Constructs

**Fig. 3:**
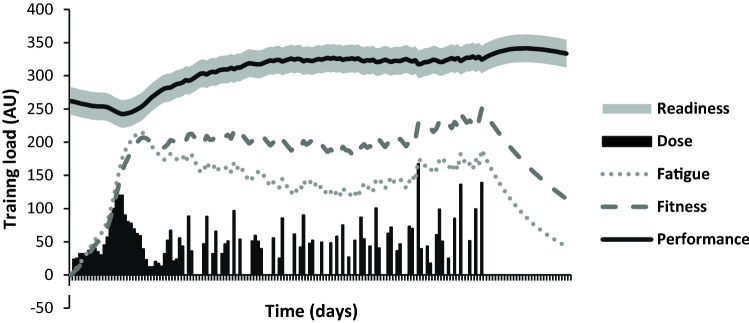
Modelled changes in performance over a training block with the inclusion of readiness as a fourth component to the FFM. *AU* Arbitrary units, *FFM* fitness–fatigue model

The FFM developed by Banister et al. [49] is a deterministic model that assumes any change in performance can be attributed solely to training [53]. However, more comprehensive reformulations of the model have been devised to feature error components [54] that acknowledge the non-training-related stressors such as sleep, nutrition, and illness that can influence performance [55]. We propose that this stochastic error component can be viewed synonymously with the concept of readiness that is often used in the descriptions and definitions of autoregulation of training [16, 21, 28, 47, 56]. Under our reformulation of the FFM, it can be said that performance on a given day is equal to the sum of training- and non-training-related components, such that:

$$p\left( t \right) = p_{0} + {\text{Fitness}}_{\varSigma } \left( t \right) + {\text{Fatigue}}_{\varSigma } \left( t \right) + {\text{Readiness}}\left( t \right) ,$$

where $p\left( t \right)$ represents an individual’s performance on the given day $t$; $p_{0}$ is the baseline performance; ${\text{Fitness}}_{\varSigma } \left( t \right)$ is the sum of the fitness components across the training sessions; ${\text{Fatigue}}_{\varSigma } \left( t \right)$ is the sum of the fatigue components across the training sessions, and ${\text{Readiness}}\left( t \right)$ is any change in performance caused by non-training-related stressors. Briefly, readiness can be conceptualised as a stochastic fluctuation in performance with a mean equal to 0 and variation equal to $\sigma^{2} .$ As shown in Fig. 3, the model can be presented graphically as a deterministic trace based on converting the training input to fitness and fatigue, which is then surrounded by a region of uncertainty which represents the non-training-related factors. From a conceptual standpoint, the integration of readiness as a component in the basic FFM highlights that adjustments to training generally need not be made unless the changes in performance differ from those that are expected as a result of the programme. In addition, we describe in Table 3 how changes in performance which are attributable to readiness on any given day could theoretically be estimated by subtracting observed performance from predicted performance (See Table 3 for definitions).

**Table 3:** Definition of key autoregulatory concepts under the proposed framework

Term	Definition
Autoregulation	An approach to exercise programming that adjusts training variable(s) based on the assessment of an individual’s performance or perception thereof
Performance	Performance is operationally defined as the sum of its constituents: fitness, fatigue, and readiness
Expected performance	Expected performance is defined as the predicted performance based on training effects (fitness and fatigue) where readiness = 0
Fitness	The positive effects on performance derived from training only
Fatigue	The negative effects on performance derived from training only
Readiness	The stochastic variation in performance that is attributable to non-training-related processes/stressors. Readiness can also be viewed as the difference between observed performance and expected performance

As previously identified in this review, a range of potentially distinct constructs central to autoregulation of training have been used interchangeably, with adjustments to training recommended on the basis of readiness [21, 45, 47, 56], fatigue [16, 45], or adaptations [7]. Under the proposed framework presented in this review, these terms are unified and can be simply viewed as different constituents of performance. Therefore, the previous descriptions of autoregulation can be seen as adjustments that are made on the basis of measuring performance, where deviations may be disproportionately affected by one or more of the constituents. For example, with an untrained individual engaging in an intense strength training programme, it is probable that rapid increases in performance will occur [57]. In such a case, adjustments made to training through measurement of performance will be influenced primarily by fitness. In contrast, for individuals with substantial training experience, with well-developed physical characteristics, who are undergoing a maintenance block, there are likely to be minimal changes in performance that are attributable to training [58]. Instead, variations in performance (and therefore any adjustments made to training) will be influenced primarily by readiness. Other examples where non-training-related factors (readiness) are likely to dominate performance changes include those of clinical populations [28]. Here, the exercise dose prescribed is often conservative and the ability to perform exercise undergoes substantial deviations primarily due to factors outside the training process [28]. Finally, for high-level athletes undergoing periods of appropriately designed overreaching, it is probable that large decrements in performance will be observed [59]. Here, any adjustments made to training (usually a reduction in volume) can be said to be based primarily on fatigue and may be implemented if performance decrements become larger than those originally anticipated.

## Application of the Framework

In this section, we present a brief overview of contemporary autoregulation practices and provide further context based on the proposed framework described within. The contemporary methods are presented based on the time-scale with which the autoregulation (measurement and adjustment) process occurs. Initially, we review autoregulation practices featuring adjustments within a single session. We then proceed to review methods that measure and adjust at the beginning of the session, and ultimately to practices that adjust at the meso- and macro-cycle level.

### Within Session Autoregulation Methods

#### Repetitions in Reserve

**Fig. 4:**
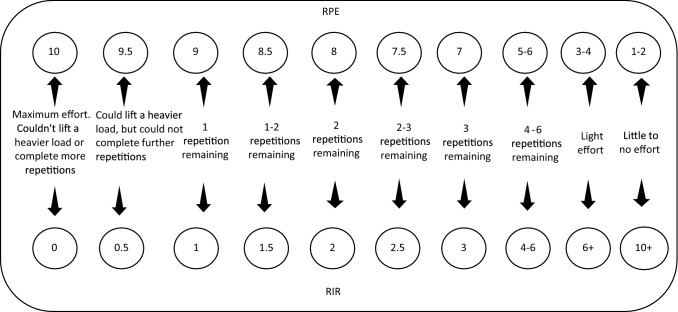
Representation of repetitions in reserve and their corresponding rating of perceived exertion values. *RIR* repetitions in reserve, *RPE* rating of perceived exertion

Currently, the most popular application of autoregulation is to adjust the exercise performed within a single session [47]. Within this time-scale, the two most popular methods of performance measurement include the repetition in reserve (RIR) scale and barbell velocity. Similarly, there exist two popular approaches to adjust exercise that include altering the load lifted or the volume performed. The RIR scale is a resistance training-specific variant of the Borg RPE scale originally devised by Tuchscherer [60], and is described more broadly as a perceptual measure of performance [45, 61]. The RIR scale provides a measure of exertion during resistance training by assessing how close an individual believes they are to momentary muscular failure [61]. Preliminary evidence for the validity of RIR-based assessment tools was first reported by Hackett et al. [62] who documented that individuals could better gauge resistance training intensity by estimating their perceived RIR in comparison to when ratings were given on the traditional Borg RPE scale. In particular, it was noted that individuals tended to provide submaximal ratings on the RPE scale even when sets were taken to momentary muscular failure [62]. Despite providing initial evidence in support of RIR-based prescription models, the results documented by Hackett et al. [62] were based strictly on an individual’s perceived RIR and, therefore, had no corresponding RPE assignment [61]. Zourdos et al. [30] later integrated these two concepts, however, and were the first to investigate the validity of Tuchscherer’s [60] proposed RIR scale (where each RPE value has a corresponding number of RIR) as a measure of resistance training intensity (Fig. 4). The results demonstrated that values given on the RIR scale were associated with proximity to momentary muscular failure as estimated by velocity during the back-squat exercise in both experienced and novice individuals [30, 61]. The RIR scale is now commonly used to autoregulate the training loads received based on daily fluctuations in performance levels [30, 61, 63]. This can be achieved by prescribing a specific RIR value and allowing the individual to self-select the load which they believe will elicit the corresponding exertion [56]. As an example, within a hypertrophy session, the autoregulation may call for 3 sets of 10 repetitions at an RIR of 2. In this case, the individual would self-select a load that they believe will result in momentary muscular failure on the 12^th^ repetition, stopping 2 repetitions short to perform a total of 10 repetitions [61]. As the session progresses, more objective adjustment criteria can be coupled with the individual’s performance during the previous sets to more accurately select a load that corresponds with the prescribed intensity [63]. Based on the performance framework outlined in Sect. 3.2 of this review, it can be seen that RIR enables the resistive load lifted to be autoregulated in line with fluctuations in an individual’s performance (i.e., maximum strength). In the case of a novice, these loads may increase on a weekly basis due to large increments in fitness. In contrast, for experienced individuals at the end of a training phase where minimal adaptations are occurring (${\text{Fitness}}_{\varSigma } \left( t \right)$ and ${\text{Fatigue}}_{\varSigma } \left( t \right)$ are balanced), autoregulation of the loads lifted based on RIR will be primarily influenced by readiness and the associated daily fluctuations caused by non-training-related stressors.

The RIR scale can also be used to systematically autoregulate sessional training volume using the so-called RIR stop points [16, 60]. In contrast to the previous example where the load was manipulated to achieve the desired stimulus, when autoregulating session volume, the load is often fixed and the number of sets (volume) adjusted to match the individual’s performance. For example, an initial load may be selected for 10 repetitions and an RIR stop point of 2 selected. If the individual was able to perform the exercise for 10 repetitions with the potential to perform at least two more repetitions, then additional sets at the same load would be performed. In contrast, if the individual upon performing the exercise does not believe that they could have performed 12 repetitions, then the particular exercise or the session may be terminated. Within the framework outlined in this review, the actual number of sets (i.e., training volume) will be autoregulated to correspond with fluctuations in performance caused by training (fitness and fatigue) and non-training-related factors (readiness). At present, there has been limited research investigating the use of RIR for the autoregulation of training volume, and that which has been conducted has been restricted to the deadlift, squat, and bench press exercises [16]. Hence, further research of RIR stop points within wider programming contexts is required.

#### Within-Session Autoregulation Methods: Velocity-Based Training

VBT is a novel method that has also become popular as a means of autoregulating both resistance training intensity and volume [21]. Training intensity is adjusted based on manipulating the load lifted within a session and the observation that a very strong inverse relationship exists between the load lifted as a percentage of an individual’s maximum and barbell velocity across a range of both upper [39, 64] and lower body [65, 66] exercises. Researchers have used this relationship to translate training programs that are commonly expressed as a percentage of an individual’s 1RM into corresponding velocity ranges [5]. For example, instead of prescribing a load of 80% 1RM, an average velocity range of 0.45–0.55 m/s may be given. In practice, a range of velocities can be prescribed that correspond with the physical quality targeted (e.g., strength or power) [21]. The individual then selects a load, such that the first repetition produces a movement velocity within the prescribed range when maximum intent is applied. In an autoregulation context, performance is the movement velocity achieved with a given load, and this will change based on fitness, fatigue, and readiness.

**Fig. 5:**
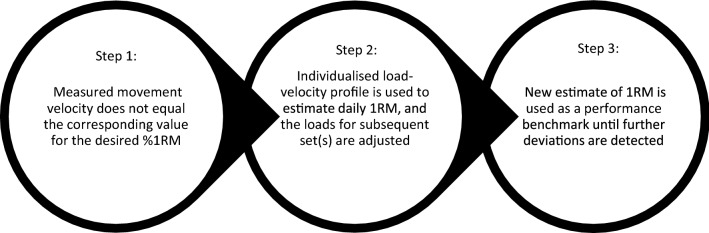
Brief overview of a process that may be utilized when seeking to regulate the prescription of resistance load on a daily and/or weekly basis. *RM* repetition maximum,  *%1RM* percentage of one repetition maximum

Jovanovic and Flanagan [67] proposed a more detailed autoregulation practice for adjusting the load lifted with movement velocity. They recommended building individualised load–velocity profiles and subsequently measuring velocity during a multi-set warm-up protocol [67]. The actual velocity obtained could then be compared with the load–velocity profile to ensure that the load lifted accurately reflected the desired  %1RM for that exercise [67]. Where this is not the case, the load–velocity profile could be used to provide an adjusted estimate of the load for subsequent sets [67]. As illustrated in Fig. 5, this adjustment process is generally achieved using the load–velocity profile to first estimate an individual’s daily 1RM, and subsequently calculating the load required to achieve the desired  %1RM. The loads used for the remainder of the session may then better reflect the individual’s current level of performance, enabling practitioners to more accurately apply the intended training stimulus.

As fatigue produces a transient decline in force generating capacity [68], movement velocity can also be associated with an individual’s level of exertion [67, 69]. As a result, this relationship can be used in the measurement of performance and the adjustment of exercise volume within a session. Two common VBT methods that have been implemented within the literature involve terminating a set when velocity decreases either by a given percentage [70, 71], or when velocity drops below an absolute value [67]. In both cases, predetermined objective criteria are used to create thresholds that bind an individual to a limited number of repetitions. In an 8-week training intervention conducted by Pareja-Blanco et al. [71], repetitions performed in the back squat were adjusted by terminating a set when velocity dropped by either 20% or 40% of the velocity obtained during the first repetition. Following completion of the intervention, the 40% group achieved greater improvements in muscular hypertrophy, whilst the 20% group achieved greater improvements in strength and power [71]. In addition, recent research suggests that there is a stable relationship between proximity to momentary muscular failure and the velocity of a movement across different exercises [72, 73]. Based on this evidence, it has been proposed that practitioners could implement absolute ‘stopping velocities’ that adjust the amount of volume performed in a given exercise or session [72]. However, at present, there remains limited evidence to support this method of autoregulation as a sustainable form of programming, with the majority of supporting evidence being cross-sectional in nature. Future research is, therefore, required to evaluate the longitudinal application of these approaches and to elucidate the specific measurement and adjustment processes (i.e., to intensity or volume) that are most appropriate for developing different physical qualities such as strength, power, and endurance.

### Meta-session Autoregulation Methods

In addition to making measurements and adjusting training within a session, an alternative autoregulation practice commonly used is to measure performance at the beginning of a session. This measurement can discern whether any modifications should be made to either specific training variables or the entire training session itself. These methods of autoregulation generally include either a direct measurement of performance, or self-report scales that provide indirect information on an individual’s perception of their performance capability.

#### Performance Measurement with the Countermovement Jump

One of the most common methods used in practice to measure an individual’s general physical performance is the countermovement jump (CMJ) [74]. CMJ performance can be measured using a broad range of kinematic and kinetic variables [75]; however, the most commonly reported variable is mean vertical jump height across multiple trials [75]. In a comprehensive meta-analysis by Claudino et al. [75], it was reported that consistent measurement of average CMJ height could be appropriately used to monitor changes in general physical performance. Whilst CMJ height has been shown to be a sensitive measure of underlying force and power capabilities on a longitudinal basis (≥ 3 weeks), it is unclear whether it provides a sensitive measure of performance change across shorter time intervals [76]. It has been reported in multiple studies that individuals may modify their jump strategy during periods of fatigue to maximise jump height despite a reduced capacity to produce force [76–79]. Each of these studies calculated jump height via the peak velocity of the centre of mass. However, it is possible that alternative methods of calculating jump height (e.g., time in the air; velocity of an attached load) may further influence the sensitivity of the measurements made [80]. Nevertheless, given the CMJ is accessible, quick to measure and can be performed daily without generating additional fatigue [74], it is a favourable candidate as a performance measurement tool within autoregulation of training [29].

A proposed method of autoregulation using CMJ performance at the meta-session level is through a threshold statistic [29]. Claudino et al. [46] recently investigated the feasibility of a novel method referred to as the minimal individual difference (MID) to autoregulate training volume. The authors recommended that athletes perform a series of eight vertical jumps over 2 days, using the data to calculate the standard deviation of jump heights around their true value. Here, true value refers to the jump height that would be achieved in a hypothetical CMJ test unaffected by measurement error [81, 82]. The standard deviation is then converted into a confidence interval that represents the MID and, therefore, a range of plausible values within which true performance resides [46, 81, 82]. A baseline measure of CMJ height is then established, and at the beginning of each subsequent session, the ability to perform is measured by performing multiple CMJs. If the observed performance and associated interval generated by the MID do not overlap the baseline measurement of performance, then either an increase or decrease in loading can be considered depending on the direction of the performance change [46]. However, practitioners wishing to implement this form of autoregulation may wish to consider the aforementioned sensitivity issues surrounding the use of CMJ height and adjust any monitoring protocols, including the methods used to calculate jump height, or the CMJ variables assessed accordingly [76]. Additionally, the practice may require modification in cases such as overreaching programs where an individual’s performance is expected to decrease substantially over the short to medium term. That is, in a well-designed overreaching program, the structure is designed to induce and manage substantial levels of fatigue. Therefore, to modify the program when the expected levels of fatigue are measured would be counterproductive.

#### Flexible Nonlinear Periodisation

The FNLP model developed by Kraemer and Fleck [38] provides an alternative method of autoregulation at the meta-session level. With this approach, athletes select from one of many possible training sessions based on either a direct measurement of performance, perceived capability to perform [9], or from a measurement based on other factors such as motivation to train [38]. In general, when performance or perceptions of ability to perform (e.g., high fitness, low fatigue, or high readiness) are elevated, the individual may select more challenging sessions. In contrast, when performance or perceptions thereof are decreased, less-challenging sessions may be selected. In many variants of FNLP, all of the training sessions scheduled are completed over the course of a macro-cycle [38]. Therefore, the purpose of FNLP is often not to change the content of a particular session, but rather to alter the distribution of training to better align with an individual’s underlying performance [17]. Colquhoun et al. [8] investigated the effectiveness of this method in well-trained individuals, enabling those in a volume and intensity matched intervention group to self-select their daily exercise session from either a strength, power, or hypertrophy focused session on a week-by-week basis. No significant differences were identified between the groups across any of the outcomes measured (bench press, squat, and deadlift 1RM).

### The Program Level

The final level of autoregulation identified in this review is the adjustment of entire programmes or training blocks based on measurement of an individual’s performance. There are presently few such methods that exist; however, we present a potential example using the FFM to demonstrate the creativity that can be used.

#### The Fitness–Fatigue Model as an Autoregulation Tool

**Fig. 6:**
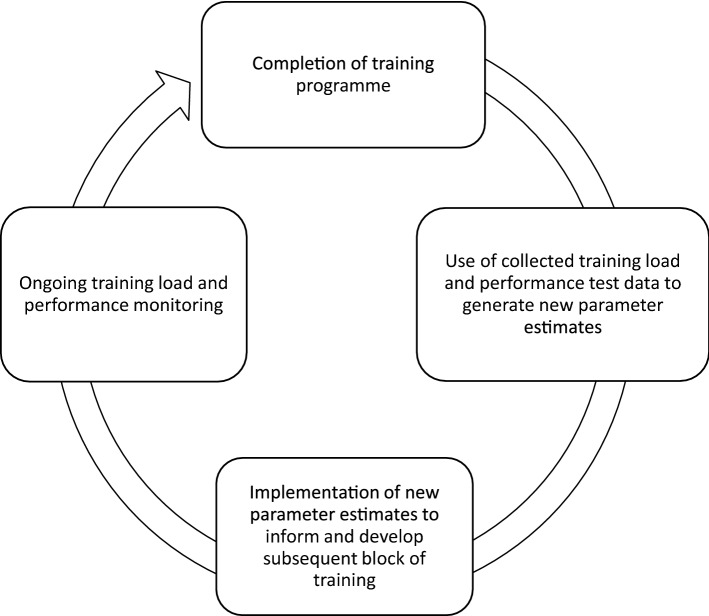
Hypothetical outline of how a fitness–fatigue model may be used to autoregulate the training received at the programme level

In Sect. 3.1 of this review, the basic FFM was introduced as a means of conceptualising and operationally defining the key constructs of autoregulation. However, it is possible to use one of the many FFM variants as an autoregulation tool in and of itself. Research has demonstrated that with appropriate training data and frequent performance measures an FFM can be fit to an individual by estimating model parameters over a sufficiently long training period [53]. The estimated parameters can be viewed as a holistic performance measurement of the individual. With the basic four parameter FFM, the measurement represents the relative magnitude of fitness and fatigue response to a given stimulus, the recovery rate of fatigue, and the stability of adaptations. With these estimated parameters, a planned training program can be simulated and adjusted if the parameters predict an alternative training program will produce superior results. It would be expected that an athlete or coach would simulate a limited number of potential training programs to inform decision-making [50]; however, research has demonstrated that sophisticated computer algorithms can develop training programs that account for a range of realistic constraints [83, 84]. Irrespective of the particular adjustments made, it is clear that the FFM and predictions could be used in autoregulation on an iterative basis (Fig. 6) where each time the measurement is made, and the FFM fit, the updated parameters provide insight into the changing dynamics of the individual and their response to training. Additionally, research has established that recursive FFMs where parameters are updated based on incoming data can be used to obtain a better model fit [85]. This approach could theoretically lend itself to a dual autoregulation scheme where an entire training block is originally devised, and smaller adjustments are later incorporated as and when required based on an iterative reassessment. This approach remains largely theoretical at present, and future research is required to appraise its effectiveness and feasibility in practice.

## Influence of Measurement Error

As outlined throughout this review, autoregulation is a two-step feedback process that individualises and adjusts training based on the measurement of performance. However, it is important to consider the various sources of measurement error that may impede the autoregulation process [4]. If adjustments are consistently made using erroneous performance values, it is probable that autoregulation will be less effective than standard practice. As all measurement comprises some degree of error, this feature should be incorporated within various autoregulation practices. For example, when performing VBT, the velocity ranges prescribed should be made wide enough to accommodate for typical error, but not so wide that the training stimulus becomes nonspecific. The technology used to measure velocity (e.g., a force plate or linear position transducer) will likely have to be consistent across sessions and possess suitable validity and reliability. Ensuring these standards will aid practitioners in accurately measuring and isolating ‘true’ changes in performance [4] and will enhance the reliability of performance estimates over time [67].

Measurement error will also be an important feature of autoregulation where threshold statistics are used to either terminate a set/session (within session level) or assist with selecting a session to perform (e.g., use of MID at the meta-session level). In the case of terminating a set/session, the individual may choose to terminate only when the velocity falls below the threshold on multiple consecutive instances. This would reduce the chance of measurement error and accompanying biological noise [82] terminating the set/session prematurely. In contrast, measurement error may be more challenging to account for when a threshold statistic is used in autoregulation at the meta-session level. For example, statistics such as that used in MID are based solely on the measurement error associated with a specific test [81, 82]. If the range used is too narrow, then this may lead to well-designed training programs being adjusted too frequently. In contrast, if the range used is too wide, this may lead to true changes in performance that warrant adjustments not being made. To avoid such issues, individuals should ensure that there is correspondence between the magnitude of confidence intervals and the true performance change upon which adjustments to training are considered appropriate.

## Conclusion

In this review, we have proposed a novel framework derived from the existing theory and models to develop a more systematic conceptualisation of autoregulation. We have suggested that autoregulation can be described as a malleable training framework within which training adjustments are made based predominantly on an individual’s performance, or their subjective assessment thereof. Using an FFM and specifying performance changes as a result of training-related processes (fitness and fatigue) and non-training-related processes (readiness), the proposed framework clarifies key terminologies and concepts that have previously been used ambiguously. This review has also highlighted that contemporary autoregulation practices can be considered across multiple timeframes (within session, meta-session, and across program level) and that adjustments can be made either to acute program variables (intensity and volume) or the distribution of training sessions when changes in an individual’s performance do not match those which are expected.

Perhaps one of the most important take-home points from our review and the framework proposed herein, is that autoregulation is highly context-specific and should be viewed as an adjunct to existing practice, rather than as an alternative or a replacement per se. What is most appropriate may be influenced by a range of factors including the setting, the individual and their goals, the experience of the practitioner, and the resources available. It is hoped that the introduction of a conceptual framework will help in highlighting general features of the autoregulation process that can assist in the synthesis of research findings, even if the specifics of the training programs investigated are different. Whilst the concept of autoregulation of training has been promoted since the 1940s, intensive research efforts have only recently begun. However, for continued development of the area, future research should propose and attempt to establish a range of general principles upon which the effectiveness and feasibility of specific practices can be identified.
